# *In situ* modified mesoporous silica nanoparticles: synthesis, properties and theranostic applications

**DOI:** 10.1039/d4bm00094c

**Published:** 2024-10-07

**Authors:** Chloe Trayford, Sabine van Rijt

**Affiliations:** a Department of Instructive Biomaterials Engineering, MERLN Institute for Technology-Inspired Regenerative Medicine, Maastricht University P.O. Box 616 6200 MD Maastricht The Netherlands s.vanrijt@maastrichtuniversity.nl

## Abstract

Over the last 20 years, mesoporous silica nanoparticles (MSNs) have drawn considerable attention in the biomedical field due to their large surface area, porous network, biocompatibility, and abundant modification possibilities. *In situ* MSN modification refers to the incorporation of materials such as alkoxysilanes, ions and nanoparticles (NPs) in the silica matrix during synthesis. Matrix modification is a popular approach for endowing MSNs with additional functionalities such as imaging properties, bioactivity, and degradability, while leaving the mesopores free for drug loading. As such, *in situ* modified MSNs are considered promising theranostic agents. This review provides an extensive overview of different materials and modification strategies that have been used and their effect on MSN properties. We also highlight how *in situ* modified MSNs have been applied in theranostic applications, oncology and regenerative medicine. We conclude with perspectives on the future outlooks and current challenges for the widespread clinical use of *in situ* modified MSNs.

## Abstract 2

This review describes *in situ* modification of MSNs and how this effects their material properties. It highlights their application in theranostics, oncology, and regenerative medicine field, concluding with future challenges for clinical use.

## Graphical abstract

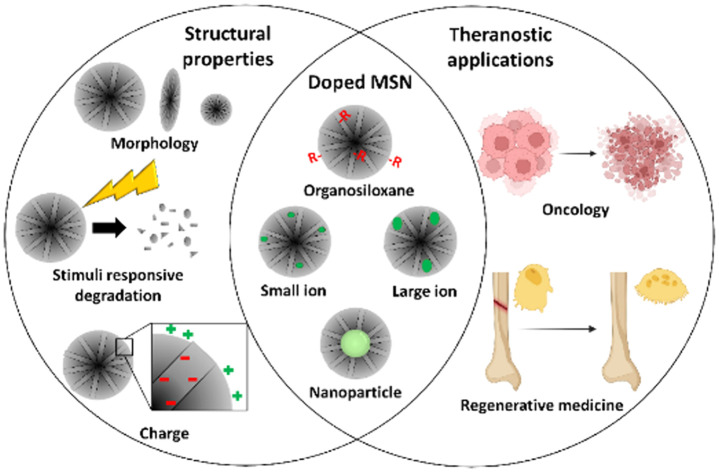

## 1. Introduction

Mesoporous silica nanoparticles or MSNs are made of amorphous, polymerized, mesoporous silicate typically in the size range of 10–200 nm. The polymerization units of MSNs are SiO_4_ tetrahedra, a covalent bonding structure where silicon and oxygen have oxidation states +4 and −2, respectively. Solid silica particles comprised of the same substance were first described as far back as 1968 in a pioneering report by Stöber *et al.*^1^ In 1992 the first examples of ordered, mesoporous silica solids formed from supramolecular surfactant arrays were discovered separately in reports by Yanagisawa *et al*. and Mobil Research and Development Corporation.^2–4^ This, coupled with the discovery of mesoporous silicates as drug and biomolecule delivery systems,^5–7^ inspired the synthesis of MSNs with ordered mesostructures in 2001 by modification of the Stöber process.^8^ MSNs exhibit an exceptionally large surface area compared to solid silica NPs due to their uniform porous structure. Fundamentally, MSN synthesis is a sol–gel process involving the assembly and hydrolysis of tetraalkoxysilane precursors (usually tetraethoxysilane; TEOS) around surfactant micelles known as structure directing agents (usually cetyltrimethylammoniumbromide) in an alcohol–water mixture with ammonia as a catalyst.

Since their discovery, many additions and iterations of the basic synthesis protocol have led to control over the chemical and physical properties of MSNs and as such, allow them to be tailored for a variety of specific applications such as sensing,^9^ catalysis^10^ and drug delivery.^11^ In particular, the Lin group pioneered a vast array of surface functionalization strategies that improved MSN drug delivery efficiency^12–14^ and catalytic performance.^15–18^ MSNs have been most popular in the biomedical field for use as drug delivery platforms. Compared to other porous nanomaterials such as porous polymer NPs, mesoporous carbon NPs and metal–organic frameworks, MSNs benefit from their ease of synthesis, stability, biocompatibility, tunable porosity, and vast functionalization possibilities.^19,20^ For example, easy and selective modification at the core, matrix or surface level has allowed MSNs to become multifunctional drug carriers.^21–24^ One such application of multifunctional MSNs is for theranostics. Applied to nanomedicine, theranostics describes the delivery of therapeutics by nanoconstructs that simultaneously provide a diagnostic readout.^25^ MSNs used for theranostics usually take advantage of the high loading capacity of the mesopores to load drugs such as antibiotics and anti-cancer drugs while imaging agents are incorporated at the core, matrix or surface.^26–28^

**Fig. 1:**
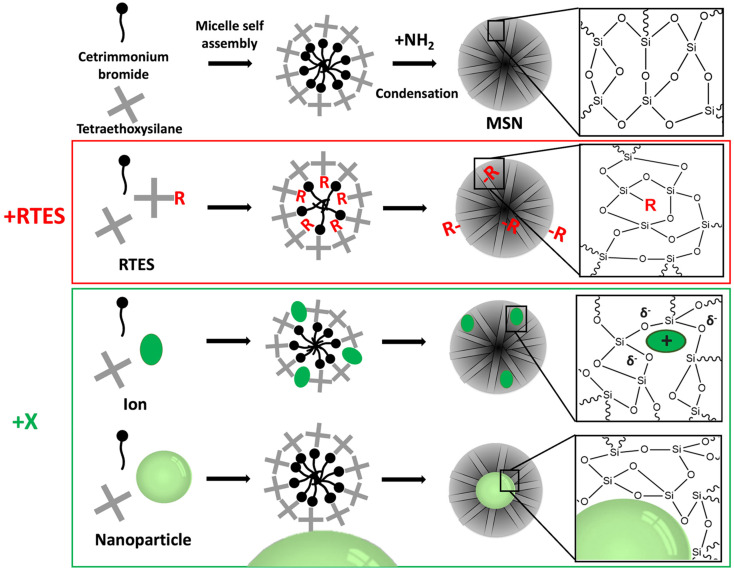
A schematic of the synthetic route of RTES and X incorporation in MSNs, showing the different methods by which dopants incorporate within the surfactant template and their eventual distribution within MSNs.

There are a vast number of strategies to create multifunctional, theranostic MSNs. For example, imaging agents such as gold or iron oxide nanoparticles (NPs) or dyes like rhodamine B or fluorescein can be encapsulated within the core of MSNs. Furthermore, antibodies, peptides, NPs or polymers can be attached to the surface to enable sensing, (sub)cellular targeting and can also act as pore gating systems.^14,29,30^ Moreover, functional groups such as amines or thiols and ions such as Ca^2+^ or Eu^3+^ can be incorporated in the silica matrix *via* co-condensation or doping, respectively, which can endow bioactivity, biodegradability, luminescence and improve drug loading within the pores. *In situ* modification of MSNs refers to co-condensation, doping and electrostatically mediated silica nucleation to incorporate groups, ions and NPs within the silica matrix during synthesis by forming ionic or covalent bonds with framework oxygen atoms or by spatial entrapment, respectively. *In situ* modification of MSNs is a convenient functionalization method that is compatible with further post-modifications. Additionally, *in situ* modification allows control over many different aspects of MSN properties such as biodegradability, size, shape, porosity, and chemical properties. Incorporated materials can be separated into two categories: organo-alkoxysilane (RTES) including (3-aminopropyl)triethoxysilane (APTES) or mercaptopropyltriethoxysilane (MPTES) and non-siloxane species (in this manuscript referred to as X) such as ions or NPs (Fig. 1). For this review, RTES refers to alkoxysilanes where an alkoxyl group is replaced by an organic ‘R’ group as well as dimerized alkoxysilanes where ‘R’ acts as the dimer bridge. While all RTES residues are incorporated in the MSN matrix by a process called co-condensation, the incorporation mechanism of X is not always known.

### 1.1. RTES co-condensation

Co-condensation with RTES involves the spontaneous co-assembly of a siloxane precursor (usually TEOS) with RTES around surfactant micelles (usually CTAB) followed by simultaneous condensation and hydrolysis which connects the precursors by siloxane bonds (Si–O–Si) (Fig. 1). Co-condensation using RTES was first reported by Burkett *et al.* Here, PTES (phenyltriethoxysilane) and OTES (*n*-octyltriethoxysilane) were injected simultaneously with the siloxane TEOS to produce MSNs with hydrophobic surfaces.^31^ Since then, many different RTES analogs’ have been developed,^32^ commonly by substitution reactions of chlorine in chlorosilanes with nucleophilic organic compounds such as amines or alcohols. As such, charged RTES analogues where R = sulphonates, phosphonates, amines or thiols and hydrophobic RTES where R = long chain aliphatic or aromatic have been easily synthesized. When incorporated throughout the matrix of MSNs by co-condensation, RTES can endow properties such as charge, hydrophobicity, degradability and magnetism.^33^ Alkoxysilanes can also be adapted before or after MSN synthesis. Modification of RTES prior to synthesis can assist in the homogeneous incorporation of dopants such as the fluorescent dyes rhodamine (RITC) and fluorescein isothiocyanate (FITC) by bonding amines of APTES with isothiocyanate in FITC or RITC to form thiourea linkages.^34^ Additionally, prior dimerization of alkoxysilanes bridged by cleavable organic groups can enable the formation of MSNs with stimuli responsive degradability.^35,36^

### 1.2. Incorporation of ions and NPs (X) in MSNs

X incorporation involves the electrostatic interaction of X with the siloxane precursor and surfactant. The size and charge of X dictate the incorporation mechanism, where X is either spatially confined in the silica matrix^37,38^ or bound with silicon (Si–X) or oxygen (Si–O–X).^39–41^ X incorporation in mesoporous silica can be attributed to the seminal work of Larry Hench where a material termed Bioglass® was created by doping with specific ratios of Na^+^, Ca^2+^ and P^5+^ ions.^42^ This has since been adopted by several groups to produce ion-doped MSNs with highly ordered mesopores.^43–45^ Mesoporous bioactive glass nanoparticles (MBGNs) are considered a separate field due to their specific SiO_2_–Na_2_O–CaO–P_2_O_5_ composition; however, MBGNs have undoubtedly paved the way for X incorporation in the MSN field. Possible materials can include ions of different sizes such as small transition metals (*e.g.* Cu^2+^, Fe^2+^, Mn^2+^ Zn^2+^, and Ni^2+^) or lanthanides (Gd^3+^, Ce^3+^, Eu^3+^, Tb^3+^, and Yb^3+^) as well as NPs such as gold NPs (AuNPs), superparamagnetic iron oxide NPs (SPIONs), silver NPs (AgNPs) and quantum dots (QDs).^46^ For ion doped-MSNs a precursor (*e.g.* CaCl_2_) is added to the reaction mixture to provide an ion in a positive oxidation state while for NP-incorporated MSNs, NPs (*e.g.* AuNPs) must be positively charged and added in the water phase. *In situ* X incorporation endows MSNs with inherent biodegradability due to inhomogeneous and weaker bonds to silicon, which compromise the structural integrity of the MSN matrix (Fig. 1). Moreover, X incorporated MSNs can be used for therapeutics, for example, when X is an ion (*e.g.* Ca^2+^) that can promote specific biological processes like directed stem cell differentiation. Additionally, when X is an imaging active material such as Gd^3+^, AuNPs and QDs, X-MSNs can be used as contrast agents in fluorescence imaging, magnetic resonance imaging (MRI) and computed tomography (CT), respectively.^38^

In this review, we discuss the synthesis of *in situ* modified MSNs and their use in imaging and therapeutic applications. In section 1, the effects of the size, charge, stoichiometry and the presence of additional modifiers on the MSN structural properties such as porosity, homogeneity, size, biodegradability and drug loading efficiency are outlined. In section 2, we summarize how *in situ* modified MSNs can be used in theranostic applications. In this review, we do not consider further (surface) modifications of *in situ* modified MSNs *e.g.* the post-synthetic attachment of ligands such as targeting agents or sensors since this is a large subfield of MSN research with many excellent recent review articles.^22,47–49^

## 2. Effect of material incorporation on MSNs’ structural properties

Dopant chemical and physical properties as well as stoichiometry and the presence of co-dopants highly influence MSNs’ morphology, degradability, and surface charge. Since MSN structural properties directly relate to cell uptake, clearance, and drug delivery, it is critical that they can be modulated for the successful use of MSNs as theranostic probes. In general, increasing material size and incorporation ratio increases MSN polydispersity. However, this can be regulated by the addition of further matrix modifiers that reduce material clustering and MSN heterogeneity. The morphological properties of MSNs such as the surface area, size and porosity are directly related to MSN degradability; a large surface area and higher porosity lead to increased rates of degradation. Degradation can also be controlled by co-condensing MSNs with alkoxysilanes, a siloxane dimer bridged by an organic group. With the use of organic groups that cleave under certain conditions, stimuli responsive degradation of MSNs is possible and enables targeted drug delivery. The efficiency of drug delivery depends on the surface properties of MSNs that facilitate drug loading/absorption. Incorporating charged/hydrophobic materials in MSNs enables a wider array of therapeutics to be loaded by non-covalent interactions. In section 2.1 we describe the factors that affect MSN formation and structural properties, in section 2.2 we explain how *in situ* modification of MSNs can be used to modulate MSN degradation and in section 2.3 we discuss how *in situ* modification can adjust the surface charge and drug loading efficiency of MSNs.

### 2.1. Morphology

**Fig. 2:**
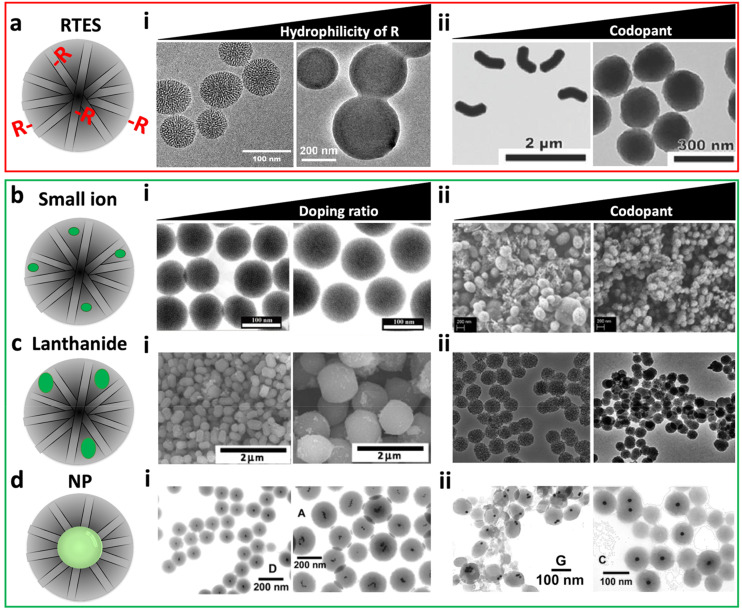
The effect of material type, chemical properties, ratio and the presence of additional matrix modifiers on the MSN morphology and structure. (a) RTES materials where (i) MSNs *vs.* APTES co-condensed MSNs which display increased size and reduced porosity. Reproduced from ref. 51 with permission from ACS, copyright 2008. (ii) 100% RTES where R = ethylene MSNs *vs.* 50%/50% RTES where R = ethylene/disulphide co-condensed MSNs. Dual RTES co-condensation reduces the size and aspect ratio of MSNs. Reproduced from ref. 56 with permission from Wiley, copyright 2014. (b) Small ion dopants where (i) 1% Cu-MSNs *vs.* 5% Cu-MSNs; increasing the Cu doping ratio increases the MSN size. Reproduced from ref. 58 with permission from Elsevier, copyright 2016. (ii) Ca-MSNs *vs.* Ca, Mg, and Sr co-doped MSNs. Co-doping reduces the size and increases the homogeneity of doped MSNs. Reproduced from ref. 59 with permission from MDPI, copyright 2021. (c) Lanthanide ion dopants where (i) 5% Eu-MSNs *vs.* 10% Eu-MSNs; increasing the Eu doping ratio increases the MSN size. Reproduced from ref. 60 with permission from Elsevier, copyright 2011. (ii) Gd-MSNs *vs.* Gd, Al co-doped MSNs. Co-doping decreases the MSN size. Reproduced from ref. 61 with permission from the RSC, copyright 2014. (d) NP incorporation where (i) high gold NP : surfactant ratio *vs.* low gold NP : surfactant ratio. Increasing the ratio of gold NP : surfactant reduces the size and gold NP clustering in MSNs. (ii) gold NP@MSNs without ethanol co-solvent *vs.* with ethanol. The presence of ethanol leads to the formation of MSNs with centrally located gold NPs with a disordered pore structure. (i and ii) Reproduced from ref. 62 with permission from ACS, copyright 2003.

The size and charge of incorporation materials have a critical impact on the MSN structure. For RTES materials, the size and charge are dictated by the ‘R’ group composition. In reports by Huh^50^ and Bein *et al.*,^51^ increasing R group hydrophobicity (R = benzyl/alkyl) stimulated the formation of smaller MSNs with sizes between 50 and 80 nm and higher aspect ratios while increasing hydrophilicity (R = amine/thiol) led to the formation of larger more spherical MSNs between 250 and 400 nm (Fig. 2a). The differences in the size and aspect ratio were explored in several studies by the Lin group who postulated that hydrophobic groups align better with organic tails of the surfactant while hydrophilic groups disrupt micelle formation.^50,52,53^ This effect only occurs if R group sizes are small. In a report by Urata *et al.* MSNs were synthesized with hydrophobic RTES residues where R = methylene, R = ethylene or R = ethynylene. As the size of the R groups increased, MSNs with higher polydispersity and diameter (20 to 100 nm) were formed.^54^ Thus, increasing the size of the hydrophobic R groups past a certain threshold leads to reduced water solubility and disrupted MSN formation. The RTES co-condensation ratio is another factor that can drastically affect the formation of MSNs by disruption of the growth mechanism. Usually, the RTES doping ratio does not exceed 10 mol% (1 : 10, R : Si).^51^ However, when MSNs are co-condensed with organo-bridge type RTES residues, homogeneous MSNs may be formed even up to 100 mol%. This is a different class of materials called periodic mesoporous organosilica NPs or PMO-NPs which exhibit regular R incorporation at a high ratio (1 : 2, R : Si), the details of which are explained elsewhere.^55^ Aside from using organo-bridged RTES, additional modifiers have been developed as another method to improve MSN homogeneity and control the morphology while imparting additional functionalities to the MSNs. In several studies by Croissant *et al*., MSNs were co-condensed with multiple RTES residues where R = ethylene and either R = phenylene or R = disulphide.^56,57^ It was found that with an increase in the ratio of disulphide^56^ or phenylene, MSNs become spherical, smaller and less porous (Fig. 2a).^57^

Non-siloxane materials (X), such as Ca^2+^, Cu^2+^, P^5+^, Sr^2+^, Mn^3+^ and Zn^2+^ with similar sizes to those of Si and O are most likely to homogeneously distribute in the MSN framework. Several studies have shown that these ions are able to interact with oxygen *via* electrostatic interactions *e.g.* SiO^−^ X^2+^.^26,39,63–65^ For example, Gu *et al*.^64^ showed that Ca^2+^ doped MSNs (Ca-MSNs) display more Q^*n*^ peaks with lower *n* values (*n* = number of Si–O bridges) by NMR, meaning that Ca^2+^ was likely incorporated into MSNs by forming ionic bonds to SiO^−^, reducing the number of Si–O–Si bridges.^66^ Further analysis of Ca-MSNs by FTIR and XPS also indicated the incorporation of Ca^2+^.^59,67^ Investigation into the incorporation of Cu^2+^ in MSNs (Cu-MSNs) similarly found that Cu^2+^ replaces Si and occupies octahedral sites.^41^ It was also found that prior conjugation of Cu^2+^ with RTES such as APTES resulted in increased doping homogeneity of Cu^2+^, suggesting that conjugation of dopants in the network forms more homogeneous MSNs. Other small ions such as Mn^2+^ and Zn^2+^ displayed similar incorporation behavior as shown by NMR^40^ and XRD.^68,69^ However, increasing doping ratios can impede the formation of MSNs, which past 5 mol% (1 : 20, X : Si), compromises the network.^41,58,70^ For Cu-MSNs, increasing the Cu^2+^ ratio from 1 to 5 mol% led to an increase in the MSN size from 150 (1%) to 230 nm (5%) as well as a 33% decrease in the pore volume and a 45% decrease in the surface area (Fig. 2b).^58,70^ At Cu doping ratios above 5%, MSNs were no longer formed.^58^ Disrupted MSN synthesis was also observed for Ca-MSNs above 5 mol%.^71^ Similar to RTES, co-doping with ions has been shown to control the doped MSN size, aspect ratio and homogeneity. In a report by Pouroutzidou *et al.*, ellipsoid Ca-MSNs became spherical, homogeneous and reduced in size by 40 to 70% when co-doped with Mg^2+^ or Sr^2+^, respectively (Fig. 2b).^59^ The size decrease was a result of co-dopants forming water adducts that release H^+^ ions, acidifying the reaction system and quenching MSN growth. Despite these changes, all studies were in agreement that the doped MSNs retained an ordered mesoporous structure as shown by distinct diffraction peaks on XRD and by a well-defined step under relative pressure in N_2_ adsorption–desorption isotherms.^58,71^

Next to small ions, larger ions specifically the lanthanides (Ln) Gd^3+^, Eu^3+^, Tb^3+^ and Ce^4+^ have been doped into the MSN silica matrix.^39,61,72–74^ This causes substantial changes to the MSN morphology such as decreasing surface area, pore volume and homogeneity (Fig. 2c). Ln incorporation was confirmed by Si–O–Si NMR peak broadening which usually only occurs because of increasing Si–O bond distance and angle. Furthermore, depending on the Ln ion size, different mol% of doping can be achieved. For example, MSNs can be doped with Eu^3+^ (Eu-MSNs) up to 10 mol% (1 : 10, X : Si)^73,75^ while for Tb^3+^ (Tb-MSNs) 8 mol% was used (1 : 12.5, X : Si).^76^ This is a result of the lanthanide contraction which increases ion field strength compared to small ions and enables greater electrostatic interaction with oxygen atoms in the siloxane network and the possibility for covalent bonding. Doping of Ln-MSNs has been quantified by ICP-MS, emerging photoluminescence at ^5^D_0_–^7^F_1–2_ and ^5^D_4_–^7^F_5–6_, as well as disappearing XRD peaks relating to Ln oxide reactants.^73,75,76^ Ln doped MSNs experience a reduction in the surface area and pore volume greater than undoped MSNs at the same doping ratio.^60,76,77^ This is a result of Ln clustering within the Si matrix and cavity coalescence occurring due to the lack of covalent bonding between silica and high-field strength Ln cations.^78^ This not only affects the homogeneity of the MSNs^79^ but also the functionality of the doped Ln such as the fluorescence properties of Eu^3+^ or the magnetic properties of Gd^3+^. For example, in Eu-MSNs or Tb-MSNs, dopant clustering is correlated with reduced luminescence due to non-radiative relaxation.^75^ While for Gd^3+^ doped MSNs (Gd-MSNs), relaxivity *(r*_1_ and *r*_2_) is drastically decreased by dipole–dipole interactions of Gd^3+^ ions in clusters.^77^ Here, co-doping also comes into play as a method to reduce clustering and improve the homogeneity and effective properties of the dopant such as the fluorescence quantum yield or MRI contrast.^80–82^ For example, Zhang *et al.* used Al^3+^ as a co-dopant in Gd-MSNs which reduced the MSN diameter by 10 nm and improved particle homogeneity and magnetic properties (Fig. 2c).^61^

Some X materials are too large to either interact or bond with atoms in polymeric silica. These consist of molecules or NPs such as solid silica (SSN), gold (AuNPs), iron (FeNPs) and quantum dots (QDs). NP incorporation in MSNs occurs by surfactant templated condensation of silica at the NP surface. Silica nucleation requires a charged and hydrophilic NP surface to interact with TEOS and the surfactant.^83^ Therefore, hydrophobic NPs are brought into an aqueous phase by surface ligand exchange, usually with the surfactant, which also acts as the structure directing agent, cetyltrimethylammonium bromide (CTAB).^84–89^ Another approach is to modify the NP surface with silane coupling agents to form a silica monolayer that acts as a nucleation site for mesoporous silica. This is especially applicable for coating vitreophobic metal NPs.^90–92^ The location of NPs within the MSN structure is dependent on the dielectric constant of the solvent mixture. For example, a solvent mixture with a low ratio of alcohol : water encourages the formation of silica oligomer micelles with a high aspect ratio and directional silica condensation so that NP@MSNs are formed with NPs located at the MSN perimeter (Fig. 2d).^83^ Increasing the alcohol (*e.g.* ethanol) ratio produces shorter micelles that pack discontinuously and form NP@MSNs with centrally located NPs and a disordered pore structure.^62^ The extent of NP clustering in MSNs, NP@MSN size and monodispersity is dependent primarily on the surfactant : silica ratio as well as the NP size and concentration.^93^ In our previous report we found that ratios of AuNPs between 3 and 5 mol% (1 : 21 to 1 : 29, X : Si) were necessary to achieve singly occupied NP@MSNs.^34^ While Nooney *et al.* investigated surfactant ratios, finding that ∼50 mol% (1 : 0.5, Si : CTAB) was optimal for creating NP@MSNs with no NP clustering (Fig. 2d).^62^ However, some NP aggregation is inevitable especially at high concentrations, due to van der Waals interactions between hydrophobic surfactant tails at the NP surface and in solution.^93^ NP flocculation also depends on size; smaller NPs aggregate at faster rates compared to large NPs due to decreased colloidal stability.^94^ Clustering of NPs in the core of NP@MSNs can decrease MSN homogeneity but improve theranostic capability, for example by enhancing photoluminescence^95,96^ or magnetism.^97^ Clustering has also enabled multiple NP types with different imaging capabilities such as fluorescent QDs and magnetic FeNPs to be incorporated in one NP@MSN construct allowing multimodal imaging.^84,98,99^ Despite their tendency to cluster, very small (<5 nm) NPs such as gold^100,101^ or palladium^102^ can also be intercalated throughout the MSN structure by *in situ* nucleation at sulphurous sites upon dual incorporation with RTES; however, confinement to nanoscale dimensions remains a challenge.^103^

It has also been possible to incorporate NPs and molecules such as dyes within the pore template.^104^ Incorporation within the pore template is achieved by self-assembly of X materials in the hydrophobic core of surfactant micelles prior to siloxane condensation. This works best for hydrophobic materials such as aromatic dyes but risks the eventual leakage of dyes and surfactants, which can lead to cytotoxicity.

### 2.2. Degradability

The biodegradability of MSNs can be altered by *in situ* modification which can be used to increase drug delivery efficiency or longevity of bioimaging to enhance theranostic function. MSN dissolution typically occurs by a three-step process involving rapid initial bulk degradation by pore expansion that slows down due to the surface re-association of dissolved silicates and subsequently follows a diffusion-controlled pathway.^105,106^ The degradation rate of MSNs depends on their synthesis method and environment. For example, MSNs with larger pores and large surface areas exhibit faster degradation rates due to the higher probability of contact with H_2_O and the increased number of active sites for hydrolysis.^107–109^ A report by Lin *et al.* also studied the influence of the environment where the presence of proteins alters the MSN degradation mechanism and increases the dissolution rate.^110^ Nevertheless, MSN degradation is relatively slow in biological fluids, with degradation rates reported for over 2 weeks.^111^ Increasing the MSN degradation rate by *in situ* modification enables controlled drug release and promotes faster tissue excretion *in vivo*, which can prevent toxic downstream effects from tissue accumulation such as inflammation.^112–114^ On the other hand, reducing the MSN degradation rate increases cellular retention which can be beneficial for diagnostic applications such as tracking the long-term bioprocesses of tissue regeneration and maturation.^115,116^

Doping MSNs with X introduces structural defects in the silica matrix that increase the degradation rate. Specifically, X doped MSNs contain weaker (Si–O–X) bonds throughout the siloxane framework which requires less energy to break than (Si–O–Si) bonds and as such increases the degradation rate of MSNs (Table 1). For example, Wang *et al.* showed that Ca, Mg and Zn doped MSNs degrade twice as fast as undoped MSNs, which was corroborated *in vivo*, where due to the renal clearance of degraded fragments, significantly less Si was observed in the vital organs of mice injected with X doped MSNs than with undoped MSNs.^117^ The cleavage of Si–O–X bonds is also quicker in the presence of acids and reducing agents that act as catalysts, a feature beneficial for selective drug delivery to cells/tissues that are acidic or reducing. Accordingly, Ca and Mn doped MSNs were found to degrade significantly faster in the acidic, GSH rich environment of tumors and exhibited enhanced anti-tumor effects compared to undoped MSNs.^37,67^ In particular, manganese oxide bonds are easily reduced by GSH and since two GSH molecules are used to degrade one Mn–O bond, Mn-MSNs are able to efficiently deplete GSH and break the redox balance of tumor cells.^37,118^ Although ion doped MSNs exhibit increased degradation rates compared under acidic and reducing conditions, they still degrade faster than undoped MSNs under neutral conditions.

**Table 1:** An overview of incorporated materials that have increased or inhibited the MSN degradation rate, their responding stimulus and the mechanism of degradation

Material	Sensitive to	Mechanism/effect on degradation	Ref.
Ions: Ca^2+^, Al^3+^, Mn^2+^, and Zn^2+^	Acidic pH and GSH	→Ion doped MSNs degrade by OH^−^ catalyzed by acids	26, 64 and 119
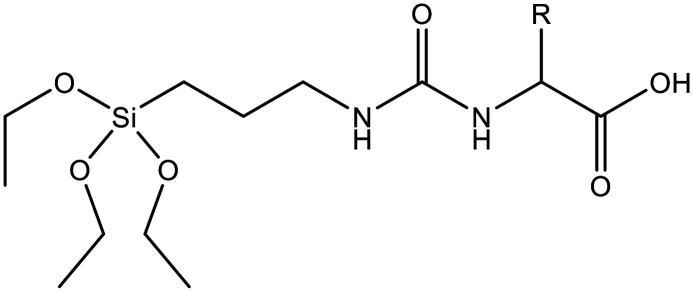	Enzymatic hydrolysis	→Urea bridged siloxane is hydrolyzed by enzymes	120
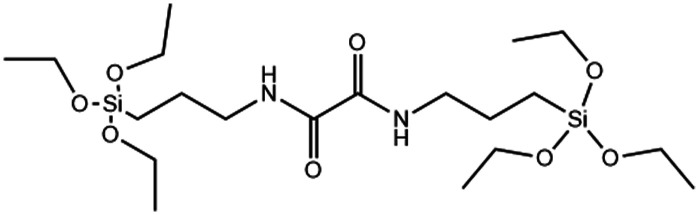	Enzyme degradable	→Oxamide bridge is cleaved by enzymes to form carboxylates and ammonium siloxanes	121
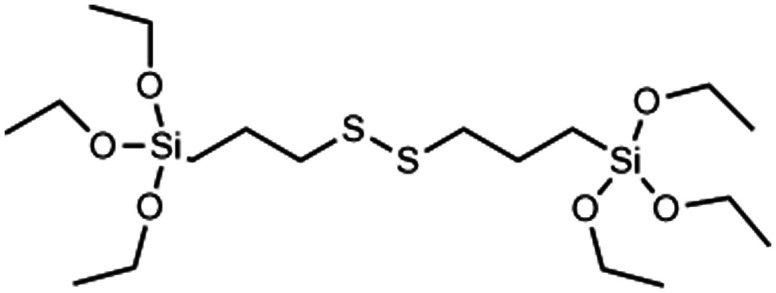	GSH	→Disulphide bridge is reduced by glutathione and other reducing agents to form thiol siloxanes	36 and 56
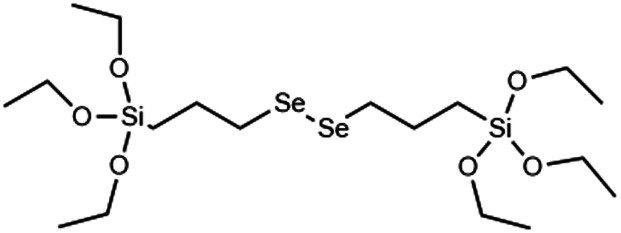	Redox (GSH/ROS)	→Diselenium bridge is cleaved by oxidation to form selenic acid siloxanes, reduction to form selenol siloxanes or X-ray irradiation	122, 123 and 124
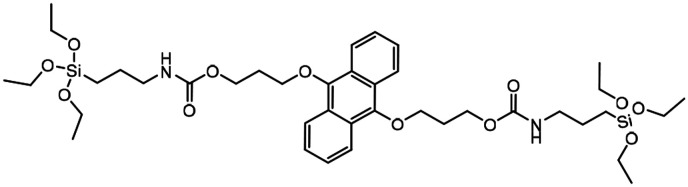	Singlet oxygen (^1^O_2_) degradable	→Anthracene bridge is cleaved by cycloaddition of singlet oxygen (^1^O_2_) to produce anthraquinone	125
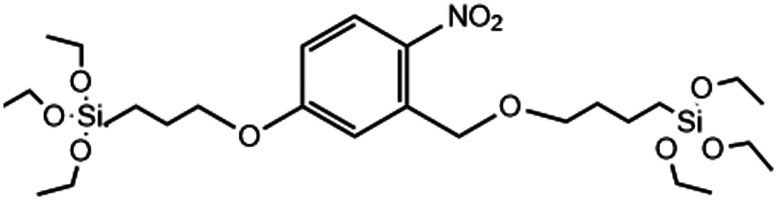	UV-light	→2-Nitrobenzyl ether bridge is cleaved by exposure to UV-light	126
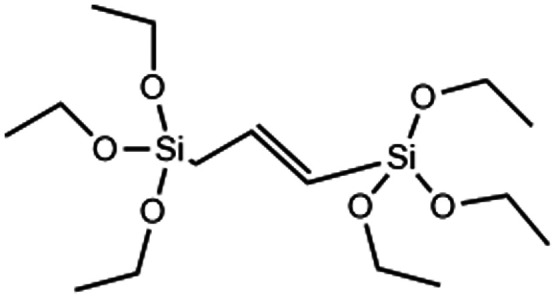	Non-degradable	→C <svg xmlns="http://www.w3.org/2000/svg" version="1.0" width="13.200000pt" height="16.000000pt" viewBox="0 0 13.200000 16.000000" preserveAspectRatio="xMidYMid meet"><metadata> Created by potrace 1.16, written by Peter Selinger 2001-2019 </metadata><g transform="translate(1.000000,15.000000) scale(0.017500,-0.017500)" fill="currentColor" stroke="none"><path d="M0 440 l0 -40 320 0 320 0 0 40 0 40 -320 0 -320 0 0 -40z M0 280 l0 -40 320 0 320 0 0 40 0 40 -320 0 -320 0 0 -40z"/></g></svg> C bridge decreases the MSN degradation rate due to increased stability compared to siloxane bonds	54

Incorporating RTES in MSNs by co-condensation can facilitate stimuli responsive degradation with RTES that are cleavable under specific stimuli such as bis(3-ethoxysilylpropyl)disulfide (BTES) which is bridged by disulfide (S–S) and reduced by intracellular glutathione (GSH). Incorporating different RTES analogs in MSNs has enabled enzyme,^127^ light,^122^ redox,^123^ temperature and pH^128^ catalyzed MSN degradation (Table 1). Thus, MSN degradation can be triggered *in vivo* both by innate differences in tissue microenvironments such as acidic, hypoxic tumors^129^ and GSH rich cell cytoplasms^130^ or by an externally applied stimulus like light, sound or heat irradiation. Spatiotemporal and time control over MSN degradation *in vivo* can improve the therapeutic efficacy of MSNs by restricting drug release to the target microenvironment.^122^

Since enzymes mostly reside in the cell cytoplasm, co-condensing enzymatically cleavable RTES analogs in MSNs is an efficient method to achieve intracellular specific MSN degradation and drug release. Enzyme degradable RTES analogs are formed by bridging alkoxysilanes with amino acid biocleavable groups such as oxamide or urea. In one study, Ratirotjanakul *et al.* created urea connected MSNs by incorporating amino acid (AA) bridged RTES residues such as glycine (Gly), aspartic acid (Asp) and cysteine (Cys).^120^ It was observed that degradation of AA-MSNs was fastest for aspartic acid doped MSNs in the enzyme containing buffer due to the presence of carboxylate anions in the side chain that acted as degradation catalysts. In a similar approach, MSNs were co-condensed with oxamide (OA) bridged RTES.^121^ These OA-MSNs only degraded in the presence of trypsin containing buffer due to the enzymatic cleavage of oxamide into carboxylate and ammonium groups.

Another popular approach for creating intracellular degradable MSNs is to incorporate RTES where R = is a GSH responsive, disulphide bridge (DIS) such as BTES (Table 1). Since cells have high intracellular (100 μM) and low extracellular (1–10 μM) GSH levels, degradation of DIS-MSNs is almost intracellular specific.^131^ In a report by Croissant *et al.* it was found that MSNs with 10, 25 and even 50% DIS degraded minimally under extracellular conditions while degradation correlated with DIS incorporation % in the intracellular environment usually over a period of 3 days.^56^ Other DIS-MSN systems loaded with drugs such as temozolomide similarly degraded and released drugs faster in cancer cells, thus displaying higher toxicity than MSNs without co-condensed RTES.^36^

Although DIS-MSNs are useful for GSH specific intracellular degradation, it has been of interest to develop MSNs capable of multiple destruction pathways to improve drug release efficiency. In this vein, RTES where R = diselenide (BTESe) has been incorporated in MSNs which can enable degradation by oxidization to release selenic acid, reduction to liberate selenol or cleavage by X-ray radiation.^123,124^ This has enabled efficient cancer therapeutics owing to the GSH and reactive oxygen species (ROS) rich cytoplasm of cancer cells and anti-cancer properties of the selenium degradation by-products. BTESe-MSNs also degrade faster than DIS-MSNs due to the lower bond energy of Se–Se (172 kJ mol^−1^) compared to S–S (240 kJ mol^−1^). Shao *et al.* found that BTESe-MSNs degraded in both 100 μM H_2_O_2_ and 5 mM GSH over ∼1 day at a rate proportional to the selenium content while DIS-MSNs degraded only under GSH conditions over ∼3 days. Furthermore, when BTESe-MSNs and DIS-MSNs were loaded with cytotoxic RNase and administered in tumor bearing mice *in vivo*, BTESe-MSN injected mice had a smaller tumor size than DIS-MSNs injected mice after 28 days at a rate inversely proportional to the selenium content.^123^ The enhanced anti-cancer effect of BTESe-MSNs was similarly observed by Chen *et al.* where BTESe-MSNs loaded with cisplatin showed significantly higher inhibition of tumor growth compared to undoped MSNs when injected in mice *in vivo.*^124^

Also, externally applied stimuli can be used to degrade MSNs. Manual control over MSN degradation and drug release mitigates influences of the biological environment. In one method, BTESe-MSNs were modified with photosensitizers, which upon light irradiation produced ROS that oxidized Se–Se bonds and degraded BTESe-MSNs. For example, Yang *et al.* reported a BTESe-MSN system co-loaded with the photosensitizer methylene blue and anticancer dug, doxorubicin (DOX), which when irradiated with red light displayed a 2.3 times increase in DOX release as a result of BTESe-MSN degradation.^122^ In another report by Peng *et al.* DOX loaded BTESe-MSNs were capped with a thermoresponsive polymer complexed with photosensitizer indocyanine green (ICG) that acted both as a DOX gatekeeper and ROS generator.^132^ Upon light irradiation, photothermal effects degraded the thermoresponsive polymer and ROS generated by ICG degraded BTESe-MSNs, increasing DOX release by 16 times compared to MSNs that were not irradiated. Despite the ∼7 times increased DOX release specificity observed by Peng *et al.* compared to Yang *et al.*, both approaches showed a significant anti-cancer effect *in vivo.* Specifically, in both approaches, tumor bearing mice injected with BTESe-MSNs and irradiated with light exhibited high levels of inflammatory cytokines and tumor growth inhibition, greater than groups without irradiation or photosensitizers.

Since BTESe-MSNs are responsive to all types of ROS as well as GSH, MSN degradation occurs in the cellular environment regardless of light irradiation. To harness complete control over MSN degradation it is useful to develop MSNs that only respond to specific ROS triggered by light irradiation. Thus, Fan *et al.* developed MSNs selectively responsive to singlet oxygen (^1^O_2_) by incorporating RTES where R = 9,10-dialkoxy-anthracene (DAA).^125^ DAA-MSNs were post-functionalized with graphene QDs which, upon exposure to UV light, produced singlet oxygen (^1^O_2_) that reacted with DAA and degraded the MSNs. Light triggered degradation of DOX loaded DAA-MSNs enabled the controlled release of DOX which was 16 times higher than the release without UV-light irradiation. This led to improved anti-cancer properties *in vivo*, where mice injected with DAA-MSNs and irradiated with UV-light showed 80% more tumor growth inhibition than mice left in the dark.

Another compelling approach has been to create MSNs that respond directly to light, eliminating the need for tedious post-modification with photosensitizers, which are also subject to *in vivo* leakage and off-target ROS production and toxicity. Accordingly, Picchetti *et al.* co-condensed MSNs with an RTES residue where R = 2-nitrobenzyl ether (NBE) that has two photocleavable ether groups (Table 1). It was found that MSNs with incorporated NBE that were loaded with cholesterol degraded upon light exposure to release cholesterol, while NBE-MSNs kept in the dark remained intact.^126^

Interestingly, MSNs have also been made more resistant to degradation by incorporating carbon double bonds into the framework. Increasing the stability of MSNs is an attractive approach for enhancing cell retention and improving diagnostic function. Ethylene bonds are less reactive and less prone to hydrolysis than siloxane bonds in undoped MSNs. In a report by Urata *et al.* RTES where R = ethylene (E) were incorporated in MSNs.^54^ It was found that the degradation of E-MSNs in PBS over 15 days was <2% compared to 90% for undoped MSNs.

### 2.3. Surface charge

The surface properties of MSNs are significantly influenced by *in situ* modification, which can be exploited to increase the incorporation efficiency of various cargo such as drugs or imaging agents. For example, incorporation of cations in MSNs increases the surface charge and loading efficiency towards negatively charged drugs, which can improve therapeutic efficacy. In a report by Choi *et al.*, Ca^2+^ doping of MSNs led to increased pore size, surface charge, and siRNA loading efficiency compared to undoped MSNs. Furthermore, when applied in the acidic cancer cell environment, Ca-MSNs degraded to liberate Ca^2+^ and SiRNA leading to significantly decreased cell survival after 24 h exposure compared to undoped MSNs.^63^ Similarly, Gu *et al.* observed that Ca-MSNs had a 5 times increased alendronate loading efficiency and subsequent higher cytotoxicity in HeLa cells compared to undoped MSNs.^64^ Enhanced loading effects have also been observed with Zn^2+^ and Ce^4+^ doped MSNs. Here, Neščáková *et al.* showed that Zn^2+^ doping significantly enhanced the adsorption of bovine serum albumin,^133^ while Ce^4+^ doping enabled a 1.3 times higher loading of the anti-malarial drug artemisinin compared to undoped MSNs.^134^ However, since only positive ions can be introduced into the MSN framework, ion-doped MSNs only facilitate active pore loading of negatively charged species.

On the other hand, RTES residues with differently charged R groups can be incorporated in MSNs and enable active loading of negative, positive as well as neutral/hydrophobic drugs. Similarly to doping MSNs with ions, RTES co-condensation with positively charged residues such as APTES (R = NH_2_) has become a widely used strategy to efficiently load negatively charged oligonucleotides in MSNs.^135,136^

Alternatively, MSNs with negatively charged surface groups such as –COOH can be prepared to allow active loading of positively charged species. In reports by Xie *et al.* MSN-COOH was formed by *in situ* modification with CPTES where R = C≡N (nitrile) followed by reduction with aqueous H_2_SO_4_.^137,138^ Carboxylic acid functionalization facilitated increased water dispersivity, DOX loading, and pH responsive DOX release, which led to increased cancer cell toxicity compared to MSNs without co-condensed CPTES. In another study, Gou *et al.* showed that poorly water-soluble anti-inflammatory drugs Nimesulide (NMS) and Indomethacin (IMC) could be loaded and released more efficiently in MSN-COOH than in MSNs. Furthermore, MSN-COOH loaded with NMS and IMC exhibited significantly decreased swelling rate, cell infiltration and inflammatory marker expression in a foot swelling model in rats.^139^

Orthogonal pore/surface functionalization of MSNs with RTES can also be achieved by the delayed co-condensation approach.^140^ This can enable different pore and surface functions such as drug trapping in the pores while the surface can improve dispersion, and be modified for tissue targeting or pore gating. For example, MSNs with hydrophobic OTES functionalized pores and a hydrophilic zwitterionic RTES functionalized surface were created to capture hydrophobic 4-heptylphenol in the pores while the surface facilitated good water dispersion.^141^ Smart gating mechanisms have also been established through the delayed co-condensation approach. Cauda *et al.* selectively decorated the outer MSN surface with –NH_2_ groups and subsequently reacted with sulfophenyl isothiocyanate.^142^ The electrostatic interaction of sulphophenyl with protonated –NH_2_ at acidic pH (∼2) sealed the mesochannels while deprotonation under neutral conditions opened the pores enabling pH dependent ibuprofen release.

## 3. Theranostic applications of *in situ* modified MSNs

Theranostics describes an approach in medicine that combines therapy with diagnostics and can drastically improve the efficacy of treatments through the personalization of care. NPs are gaining attention as possible theranostic agents as they can be tailored for specific disease profiles. Ideal theranostic nanoprobes can target cells of interest, deliver sufficient therapeutics while providing a diagnostic readout, and be effectively cleared from tissues after use. *In situ* MSN modification (section 1) is an effective strategy to create theranostic agents by introducing diagnostic capability and degradability into the MSN matrix while retaining a porous structure for loading therapeutics and a modifiable surface for attaching targeting agents. Usually, *in situ* incoporated materials are imaging agents such as ferromagnetic ions, fluorescent dyes and metallic NPs to enable MSNs to be detected by MRI, ultrasound (US), and optical or photoacoustic imaging (PAI). As such, they have been researched as theranostic tools in oncology and regenerative medicine. In this section, we describe representative examples of the use of theranostic MSNs in these two fields. In section 3.1 we describe the use of MSNs in oncological applications and in section 3.2 we discuss how *in situ* modified MSNs can be used for tissue regeneration.

### 3.1. Oncology

Oncology is a branch of medicine dealing with the prevention, diagnosis, and treatment of cancer. Theranostics is revolutionizing cancer treatment by providing crucial information to clinicians about tumor localization and size while assessing treatment effectiveness to allow the adoption of therapeutic approaches for individual cases. Since *in situ* modified MSNs have been reported to target tissue, carry different sizes of cargo (small molecule chemotherapeutics, oligonucleotides, and proteins), exhibit tumor specific drug release and degradation, and accumulate in tumors by the enhanced permeability and retention (EPR) effect, they can act as ideal cancer theranostic (chemotheranostic) agents. A wide range of materials have been used to create MSNs for chemotheranostics including MRI active agents such as Mn^2+^, Gd^3+^ and superparamagnetic iron oxide nanoparticles (SPIONs) or optical agents such as fluorescent NPs and luminescent ions.

#### 3.1.1. MSNs for MRI

**Fig. 3:**
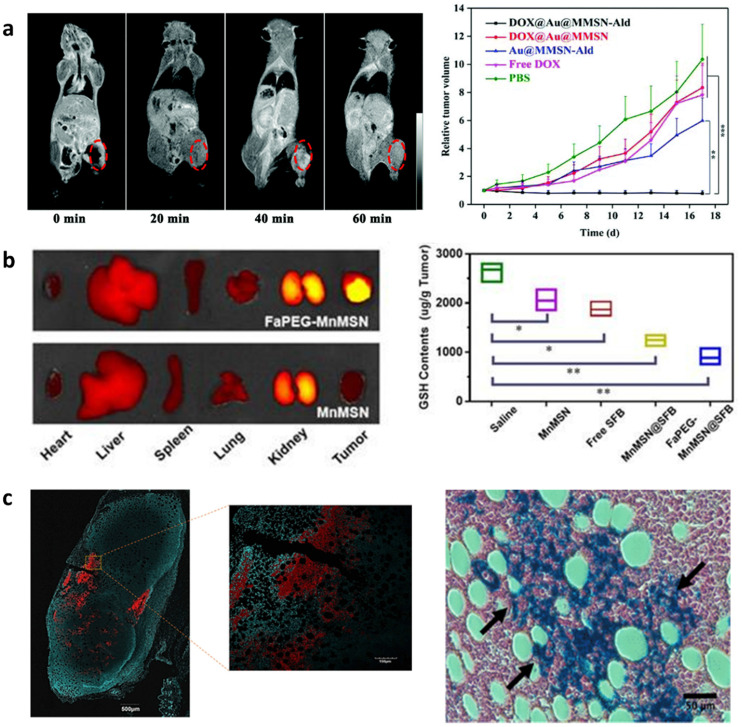
*In situ* modified MSNs as theranostic agents in oncology. (a) Cy5.5-labeled FaPEG-MnMSNs used for targeted *in vivo* fluorescence imaging of HepG2 tumors (left) and scavenging of GSH (right) in mice leading to tumor reduction and suppression. Reproduced from ref. 37 with permission from IIP, copyright 2020. (b) DOX@Au@Mn-MSN-Ald shown as simultaneous MRI contrast agents (left) and efficient tumor inhibitors (right) when injected in 143B tumor-bearing mice. Reproduced from ref. 38 with permission from the RSC, copyright 2021. (c) SPION@MSNs used as combined confocal imaging (left) and anti-tumor (right) agents when injected *in vivo* in mouse tumors followed by AMF stimulation. Reproduced from ref. 150 with permission from ACS, copyright 2018.

Manganese ion doped MSNs have become popular agents in oncology since Mn^2+^ ions are themselves chemotheranostic; they are ferromagnetic, facilitating MRI imaging (*T*_1_ weighted) but can also scavenge glutathione to induce cancer cell apoptosis (Fig. 3a).^38,143–145^ In one approach, Tang *et al.* described Mn^2+^ doped MSNs (Mn-MSNs) loaded with sorafenib.^37^ It was found that degradation of Mn-MSNs led to the simultaneous release of Mn^2+^ ions and sorafenib, which scavenged glutathione and inhibited glutathione biosynthesis, respectively. The reduction in cellular glutathione of HepG2 cells following 24 h of incubation with Mn-MSNs led to apoptosis of 36% of the cell population. Furthermore, Mn-MSNs injected in the tail vein of mice reduced tumor volume by 97% after 22 days. Acid catalyzed Mn-MSN degradation (section 2.2) and Mn^2+^ liberation also led to enhanced *T*1 weighted MRI contrast specific to tumor microenvironments. For example, the MRI signal at the hepatocellular carcinoma tumor site was observed to continuously increase over 4 h after Mn-MSN injection in the tail vein of mice and resulted from accumulating Mn^2+^ ions (Fig. 3b). In other approaches, Mn-MSNs were pore loaded with drugs to allow tumor-specific MRI imaging and simultaneous delivery of the chemotherapeutic agents DOX^26,145^ or temozolomide.^146^ Doping with ferromagnetic ions and loading chemotherapeutic drugs has become a popular approach in cancer theranostics that is also extensively described with Gd^3+^ doped MSNs.^74,147–149^

Similarly to Mn^2+^, SPIONs have been used as chemotheranostic agents. SPIONs are MRI active (*T*_2_-weighted) and vibrate in the presence of an alternative magnetic field (AMF) to enable magnetic hyperthermia. Since tumoral tissues are highly sensitive to temperature changes, magnetic hyperthermia is tumor selective.^151^ Wang *et al.* developed SPION incorporated MSNs (SPION@MSNs) in different morphologies as gene therapy agents that induce cell suicide to treat hepatocellular carcinoma.^152^ These SPION@MSNs were loaded with herpes simplex virus thymidine kinase/ganciclovir and upon application of AMF facilitated a temperature increase to 46 °C, which both enhanced gene transfection through rousing dormant cells and apoptosis by magnetic hyperthermia. Furthermore, when applied *in vivo*, SPION@MSNs could be monitored by MRI and showed the highest tumor inhibitory effects compared to NP groups without loaded gene therapeutics or AMF. Applied magnetic fields were also used to increase the tumor retention of SPION@MSNs and therapeutic performance. The magnetic-responsive properties of SPION@MSNs have also been harnessed to control drug release from the mesopores. Guisasola *et al.* showed that when DOX loaded SPION@MSNs were capped with a thermoresponsive polymer and exposed to AMF, the release of DOX could be controlled by heat-mediated polymer degradation (Fig. 3c).^150^ SPION@MSNs administration in mice followed by AMF application, led to high tumor growth inhibition after 48 h, indicating AMF specific release of DOX. Furthermore, RITC labelling of these SPION@MSNs allowed their simultaneous detection in tumor slices by confocal imaging.

#### 3.1.2. MSNs for optical imaging

MSNs modified *in situ* with optical and photoacoustic agents such as the luminescent lanthanide ion; Eu^2+^,^153,154^ up-conversion NPs (UCNPs)^155^ or gold NPs^156^ and fluorescent dye conjugated RTES residues^157^ have been extensively researched in cancer theranostics. A popular strategy incorporates light absorbing agents in MSNs and conjugates or traps photosensitizers at the surface or in the pores to enable combined optical imaging and photodynamic/photothermal therapy (PDT/PTT).^155,156^ For example, Lv *et al.* created MSNs incorporated with UNCPs (UCNP@MSNs) that also trapped photosensitizer ICG in the mesochannels by surface capping with solid silica (TEOS).^155^ UCNPs are fluorescent probes doped with rare earth ions that transfer long light wavelengths into short wavelengths. Lv *et al.* showed that NIR irradiation of ICG trapped UCNP@MSNs enabled both PA and PTT due to the non-radiative relaxation of ICG. Specifically, a 12 °C temperature increase was observed upon NIR irradiation that killed cancer cells *in vitro*. Furthermore, up-conversion luminescence and PAI facilitated UCNP@MSN visualization at a depth of 1.5 cm through mouse skin and chicken tissue. Similarly, Ning *et al.* developed gold nanostar core–shell MSNs (GNS@MSNs) that trapped ICG in the mesopores using a calcium silicate shell.^156^ These GNS@MSNs demonstrated combined PAI and fluorescence imaging that facilitated 3D imaging as well as PTT that inhibited tumor growth in a breast cancer model in mice.

### 3.2. Regenerative medicine

Regenerative medicine focuses on harnessing the body's natural repair process to regenerate and repair diseased tissues. Commonly researched strategies for tissue regeneration include cell, gene and small molecule therapy as well as biomaterial and engineered tissue implantation. Theranostic agents enable real-time monitoring of regenerative processes, which can improve outcomes through early detection of issues, reduced side effects and the personalization of treatment methods. MSN based theranostic agents are particularly suited for regenerative medicine as they can be surface functionalized to allow integration into biomaterials, target tissues or control drug release. Furthermore, doping MSNs with bioactive ions such as Ca^2+^, Zn^2+^, Ag^+^ and Sr^2+^ or loading with growth factors can improve tissue regeneration by inducing or promoting stem cell differentiation towards specialized cells to guide new tissue formation. Moreover *in situ* modification of MSNs can introduce anti-bacterial and anti-inflammatory properties that prevent infection and inflammation at the implantation site and lead to improved treatment outcomes.

**Fig. 4:**
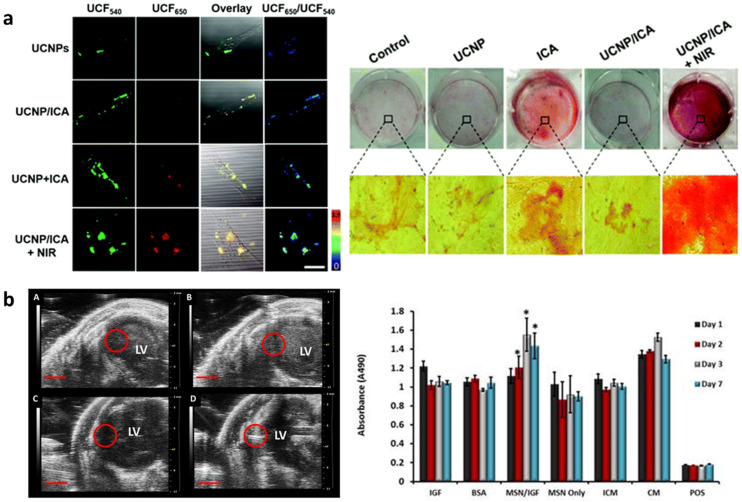
*In situ* modified MSNs as theranostic agents in regenerative medicine. (a) UCNP@MSN-azo-peptide-BHQ-3 enable real time detection (left) and induction (right) of osteogenic differentiation after uptake in MSCs and NIR irradiation. Reproduced from ref. 27 with permission from the RSC, copyright 2020. (b) Gd-FITC-MSN-IGF internalized in MSCs can be detected by MRI (left) after injection in mice as well as increase MSCs’ metabolic activity (right). Reproduced from ref. 164 with permission from IIP, copyright 2015.

Interestingly, the degradation products of MSNs (silicic acid, Si(OH)^4^) can also be used to promote tissue formation. For example, Si containing molecules have been shown to inhibit adipogenesis but can promote angiogenesis and osteogenesis through several cell-signaling pathways such as the Wnt/β-catenin and VEGF-A/VEGFR-2 pathways.^158,159^ As such, *in situ* modified, degradable MSNs are popular agents in bone regeneration and wound healing.^58,160^ Furthermore, MSNs have been used for tracing long-term regenerative processes such as cell differentiation that usually occur over weeks or months.^161,162^ In one approach, UCNP@MSNs have been used to induce osteogenesis and trace cells by fluorescence imaging. Here, UCNPs were used not only to endow MSNs with imaging properties but also to assist in the controlled release of growth factors from the mesopores to induce differentiation. Wang *et al.* described osteoinductive drug icariin loaded UCNP@MSNs which were pore functionalized with azo and modified at the surface with cell targeting peptide RGD and an osteogenesis sensor (Fig. 4a).^27^ The sensor was composed of a black hole quencher connected to MSNs by a peptide sensitive to MMP-13; an enzyme produced during osteogenesis. First, RGD facilitated the uptake of UCNP@MSNs in MSCs and, upon NIR light irradiation, pore-conjugated azo isomerized to release and disperse  icariin that induced MSC differentiation to osteoblasts. During osteogenesis MMP-13 was produced which cleaved the peptide linkage at the surface of UCNP@MSNs, leading to the expulsion of the black hole quencher and the recovery of UCNP@MSN fluorescence at 650 nm. Thus, UCNP@MSNs were not only able to trace MSCs through UCNP fluorescence at 540 nm but also monitor osteogenic differentiation in real time. UCNP@MSNs have also been used to induce chondrogenic differentiation. Yang *et al.* reported a UCNP@MSN system capable of photoinduced chondrogenic differentiation and simultaneous tracing of resulting chondrocytes. Here, UCNP@MSNs were conjugated to the photosensitizer azobenzene (azo) and loaded with chondrogenic growth factor kartogenin (KGN).^163^ The incorporated UCNPs converted near infrared light to both UV and red light, which was used to stimulate azo to isomerize, releasing KGN and enabling fluorescence imaging, respectively. When UCNP@MSNs labelled mMSCs were mixed with a 3D vitrogel in an *in vivo* implantation mouse model, chondrogenic differentiation was induced by NIR irradiation. UCNP@MSNs could also be traced *in vivo* for up to 21 days after inducing differentiation, enabling monitoring of cell migration, distribution, and engraftment.

Doping MSNs with osteogenic and adipogenic ions such as Ca^2+^, Sr^2+^, Co^2+^ and Zn^2+^ or with pro-inflammatory ions such as Eu^3+^ and Cu^2+^ can also enhance tissue regeneration.^58,73,160^ Controlled inflammation aids in the regenerative process by promoting the formation of new blood vessels (angiogenesis) that can bring oxygen, nutrients, bone forming cells and cartilage to the injury site. In a report by Shi *et al.* Eu-MSNs were developed and exposed to macrophages stimulating the production of inflammatory cytokines that were harnessed to promote osteogenesis in MSCs and angiogenesis in human umbilical vein endothelial cells (HUVECs).^73^ This was corroborated *in vivo* in both a rat cranial defect model and a chronic skin wound mouse model. It was observed that significantly more bone was formed in rats treated with Eu-MSN compared to MSN after 12 weeks. In a large wound *in vivo* mouse model, a significantly denser capillary network and smaller wound size were observed for groups treated with Eu-MSNs after 13 days. Furthermore, the luminescence of Eu^3+^ ions allowed Eu-MSNs to be detected by fluorescence imaging enabling their use as theranostic agents.

Although inflammation can aid in tissue regeneration; particularly in osteo- and angiogenesis, too much can cause cell death. Accordingly, *in situ* modified MSNs have also been developed as agents to regulate inflammation and assist in tissue regeneration.^165–168^ In a study by Li *et al.* MSNs were doped with Mn^2+^ to instill ROS scavenging and MRI imaging capabilities, then coated with a platelet membrane to enable targeting of inflamed sites.^167^ Mn-MSNs displayed a ROS and pH dependent degradation and Mn^2+^ ion expulsion profile that correlated with increased ROS scavenging ability and MRI signal. Furthermore, when Mn-MSNs were injected *in vivo* in an acute liver failure mouse model they were able to selectively target the liver and display a localized MRI signal with intensity correlated to inflammation severity. After two daily treatments with Mn-MSNs, the MRI liver signal was similar to that of healthy mice, indicating a reduction in ROS to healthy levels to aid liver regeneration.

Besides administering theranostic doped MSNs and other synthetic biomaterials, stem cell therapy is an important method to promote *in vivo* tissue regeneration.^169^ However, ensuring stem cell survival and directing differentiation post-transplantation is a significant challenge. As such, *in situ* modified theranostic MSNs can be used in combination with stem cells to improve therapy efficacy.^164,170,171^ For example, Kempen *et al.* developed a pro-survival drug, insulin-like growth factor (IGF) loaded MSNs, which was also doped with Gd^3+^ and FITC-APTES to enable fluorescence, MRI and ultrasound imaging (Fig. 4b).^164^ The developed Gd-FITC-MSNs were taken up by MSCs, degraded under intracellular conditions within a month and displayed a concentration dependent US and MRI signal. Furthermore, when Gd-FITC-MSN labelled MSCs were cultured under serum free conditions (simulating *in vivo* necrotic regions) a 40% increase in viability compared to MSCs cultured in complete media was observed after 1 week.

## 4. Conclusion and outlook

Theranostics is a medical strategy that can enable safer, more efficient disease treatment compared to traditional techniques by continuous therapy monitoring and care personalization. It is a particularly pivotal approach in oncology and regenerative medicine where disease profiles are complex and person specific. Rigorous research efforts over the last few decades have elucidated the modification possibilities of MSNs enabling them to become multifunctional agents. Specifically, *in situ* modification has propelled the use of MSNs in theranostics by endowing the matrix with imaging properties, bioactivity, charge and degradability while allowing post-synthetic modifications and pore loading. With this review we provide a relevant summary detailing the effect of various *in situ* modification strategies on the physiochemical properties and theranostic capabilities of MSNs. We highlight how *in situ* modification affects the MSN morphology, charge and degradability (section 2). We discuss that for successful *in situ* modification and to control the MSN morphology, careful consideration of the material type, ratio, and the presence of additional modifiers and solvents is necessary (section 2.1). We also describe how *in situ* modification can be used to strategically adjust MSN degradability and surface charge in order to tailor MSNs for specific applications (sections 2.2 and 2.3). Furthermore, we discussed several examples where MSNs were optimized for theranostics in oncology and regenerative medicine (section 3). A common approach uses materials with imaging capabilities (UCNPs, GNS, and Gd^3+^) to introduce MRI, US, PAI or fluorescence properties into MSNs. However, it has also been possible to use materials with inherent theranostic ability (SPIONs, Mn^2+^, and Eu^3+^) to circumvent the need for MSN post-modifications (section 3).

Despite the huge potential of *in situ* modified MSNs as well as positive outcomes in pre-clinical studies (section 3), clinical translation remains a challenge. Until now, only *in situ* modified solid silica NPs^171–173^ and micron-sized MBGs^172^ have been tested in human clinical trials. For example, bioglass particles termed ‘PerioGlas®^’^ authorized in the European market in 1995 have been implemented in over 20 clinical trials for bone regeneration in periodontal disease.^174^ In these studies, PerioGlas® significantly increased the density and volume of bone at intrabony defect sites compared to surgical debridement.^175–177^ Silica particles have also been evaluated as effective diagnostic agents. In several phase 1 clinical trials, fluorescent core, silica shell NPs known as ‘Cornell dots’ were shown as safe positron emission tomography (PET) contrast agents for effective tumor imaging.^172,173^ Although promising in the field, the size and composition of Cornell dots (6 nm solid silica NPs) and PerioGlas® (90–170 μm MBG particles) are significantly different from those of *in situ* modified MSNs, each with distinct safety profiles and *in vivo* processing mechanisms. Since *in situ* modification has a significant impact on the morphology, charge, degradability and bioactivity of MSNs, a new *in vivo* risk assessment must be carried out with each design.

In addition, much is still unknown about how to enhance *in situ* modified MSNs for theranostic applications. Optimal theranostic agents should overcome biological barriers, target specific locations, image at high resolution, and be able to controllably deliver both small drugs and large biomolecule therapeutics *in vivo*. Currently, MSN designs for theranostics focus on modifying the matrix and surface with imaging agents and delivering therapeutics by the mesopores. However, the delivery of large biomolecules such as DNA, RNA and proteins using MSNs is restricted by the small size of the mesopores (∼2–6 nm). Furthermore, incorporating multiple contrast agents in MSNs to enable high resolution imaging usually requires a series of synthetic steps that increase their batch-to-batch variability and limit clinical use. Similarly, MSN tissue targeting and controlled therapeutic delivery are typically only achieved with a number of expensive, laborious post-synthetic modifications. As such, manufacturing MSNs at a large scale remains a challenge which makes bench to bedside transition slow.

Expediting the clinical translation of *in situ* modified MSNs requires optimization and standardization of MSN designs. As summarized in section 2 of this review, reaction stoichiometry, material pre-treatment, and the presence of additional modifiers drastically affect the formation of *in situ* modified MSNs. Some general design rules, such as maximum molar ratios for ions = 5 mol% (1 : 20, ion : Si), RTES = 10 mol% (1 : 10, RTES : Si), bridged RTES = 100 mol% (1 : 1, bridged RTES : Si) and NP = 5 mol% (1 : 20, NP : Si), appeared universal amongst the investigated literature studies. Moreover, generally speaking, the homogeneity of ion or RTES incorporated MSNs could be improved by introducing additional modifiers such as smaller ions or hydrophobic RTES in the silica matrix, respectively. While for NP@MSNs the use of a co-solvent (usually ethanol at 40 mol%) (1 : 2.5, ethanol : water) is crucial to attain MSNs with centrally incorporated NPs. It is important to formulate general design rules such as the ones described here so that *in situ*-modified MSN synthesis can be standardized for the desired morphology, charge and degradability outcomes and fasten clinical applications.

Furthermore, as described in section 2.3, the number of synthetic steps needed for sophisticated MSN designs can be minimized by incorporating multiple materials *in situ* simultaneously, which is encouraging for their clinical development. For example, co-condensing MSNs with non-degradable ethylene bridged alkoxysilanes and luminescent Eu^3+^ improved cell tracing longevity compared to Eu-MSNs. Additionally, delayed material incorporation to integrate multiple functionalities at specific locations in MSNs is a promising approach for creating multifunctional NPs in a single synthesis. This would allow the creation of multimodal MSNs capable of high-resolution imaging by, for example, incorporating NPs (SPIONs, gold NPs, and UCNPs) in the MSN core while fluorescent alkoxysilanes (FITC-APTES and RITC-APTES) are co-condensed throughout the matrix. Furthermore, imaging agents can be incorporated alongside morphological adaptations, such as pore enlargement or core hollowing, to enhance the capacity of MSNs for large biomolecule therapeutics. Thus, one-pot synthetic procedures to create multifunctional MSNs are likely to increase reproducibility and hasten clinical translation.

Although *in situ* modified MSNs have been primarily used as theranostic agents in oncology and regenerative medicine, they could easily be optimized for a vast number of medicinal fields such as wound healing, vaccines and infectious diseases. For example, incorporating antimicrobial silver NPs in MSNs and pore loading antibiotics could allow combinatory treatment of bacterial infections as well as active diagnostics due to the optical properties of silver NPs. In conclusion, *in situ* modified MSN designs for theranostic clinical use have the potential to transform not only oncology and tissue regeneration but also personalized medicine as a whole, significantly improving treatment outcomes of patients worldwide.

## Author contributions

C. Trayford: conceptualization, collation of data, writing – original draft, revising and editing. S. Van Rijt: funding acquisition, project administration, supervision, editing and revising.

## Data availability

No primary research results, software or code have been included and no new data were generated or analysed as part of this review.

## Conflicts of interest

The authors declare that there are no competing financial interests or personal relationships that influenced the work reported in this review.
